# Small Substrate Transport and Mechanism of a Molybdate ATP Binding Cassette Transporter in a Lipid Environment*

**DOI:** 10.1074/jbc.M114.563783

**Published:** 2014-04-10

**Authors:** Austin J. Rice, Alistair Harrison, Frances J. D. Alvarez, Amy L. Davidson, Heather W. Pinkett

**Affiliations:** From the ‡ Department of Molecular Biosciences, Northwestern University, Evanston, Illinois 60208,; § Center for Microbial Pathogenesis, The Research Institute at Nationwide Children's Hospital, Columbus Ohio 43205, and; ¶ Department of Chemistry, Purdue University, West Lafayette, Indiana 47907

**Keywords:** ABC Transporter, ATP, Electron Paramagnetic Resonance (EPR), Lipids, Membrane Transport, Nutrient Uptake

## Abstract

Embedded in the plasma membrane of all bacteria, ATP binding cassette (ABC) importers facilitate the uptake of several vital nutrients and cofactors. The ABC transporter, MolBC-A, imports molybdate by passing substrate from the binding protein MolA to a membrane-spanning translocation pathway of MolB. To understand the mechanism of transport in the biological membrane as a whole, the effects of the lipid bilayer on transport needed to be addressed. Continuous wave-electron paramagnetic resonance and *in vivo* molybdate uptake studies were used to test the impact of the lipid environment on the mechanism and function of MolBC-A. Working with the bacterium *Haemophilus influenzae*, we found that MolBC-A functions as a low affinity molybdate transporter in its native environment. In periods of high extracellular molybdate concentration, *H. influenzae* makes use of parallel molybdate transport systems (MolBC-A and ModBC-A) to take up a greater amount of molybdate than a strain with ModBC-A alone. In addition, the movement of the translocation pathway in response to nucleotide binding and hydrolysis in a lipid environment is conserved when compared with in-detergent analysis. However, electron paramagnetic resonance spectroscopy indicates that a lipid environment restricts the flexibility of the MolBC translocation pathway. By combining continuous wave-electron paramagnetic resonance spectroscopy and substrate uptake studies, we reveal details of molybdate transport and the logistics of uptake systems that employ multiple transporters for the same substrate, offering insight into the mechanisms of nutrient uptake in bacteria.

## Teaser

**Background:** Multiple ABC transporters work in concert to transport the same substrate.

**Results:** MolBC-A allows for additional Mo uptake in periods of high external molybdate concentration.

**Conclusion:** Utilizing the established transport mechanism, molybdate uptake is concentration-dependent.

**Significance:** Our studies address the impact the lipid environment has on the mechanism of MolBC-A as well as the role this transporter plays in molybdate uptake.

## Introduction

ABC^2^ importers bring vital substrates across the cellular membrane into the cytoplasm. These transporters play a critical role in nutrient and metabolite uptake, transporting a variety of nutrients including peptides, metals, ions, and amino acids necessary for viability. All ABC importers have similar architecture: the transmembrane domain that forms the translocation pathway and the nucleotide binding domain that binds and hydrolyzes ATP, driving the active transport of substrate through the transmembrane domains (1). For ABC importers, substrate specificity is facilitated by the substrate binding protein that binds to and delivers substrate to its cognate transporter (2). The mechanism of transport varies depending on type of importer (I or II) and size of substrate (3, 4).

ABC transporters are embedded in the plasma membrane and the effect of the lipid environment on transporter function can range from subtle to essential. For purified ABC exporters, ATP hydrolysis rates are greatly stimulated by lipid addition and liposome reconstitution (5, 6). In contrast, substrate-independent activity decreases upon reconstitution for the Type II vitamin B12 importer BtuCD (7). The Type I maltose importer MalFGK_2_-MBP also loses substrate-independent activity when reconstituted from detergent to lipid (8); however, substrate-dependent conformation changes at the nucleotide binding domain appear to be qualitatively similar in detergent micelles and in proteoliposomes (9). A lipid bilayer can affect conformation change in membrane proteins by adding to (or taking from) the energetic cost of the assumed conformation. In some cases the effect of lipid is nonspecific, depending on the deformation energy of the lipid bilayer surrounding the protein (10). This nonspecific effect of lipid on protein facilitates the activities of certain toxins (11) and can promote membrane protein dimer formation (12) and cluster similar proteins (13, 14). Alternatively, site-specific lipid-protein interactions can alter the energetics of a conformation change (15) or the binding of substrate (16). Given the effect a lipid environment can have on transporter conformation and activity, the present work was conducted on a molybdate importer from *Haemophilus influenzae,* MolBC-A, in the presence of lipids (17).

*H. influenzae* is a Gram-negative human pathogen that commonly infects the lower respiratory tract (18). Nontypeable strains of *H. influenzae* lack capsular antigens commonly targeted by vaccination and currently pose a particular threat to children and persons with chronic obstructive pulmonary disease (19–21). New drug targets would be helpful in controlling *H. influenzae,* and a detailed understanding of substrate uptake and regulation may reveal such targets as well as their relative importance to substrate homeostasis.

Previous studies conducted on the MolBC-A importer indicate that the substrate binding protein MolA binds molybdate and the structurally similar oxyanion tungstate (22). Molybdate is a vital cofactor found in the active site of several enzymes involved in carbon and nitrogen cycling (23). Due to its overall architecture and high basal levels of ATP hydrolysis, MolBC-A was classified as a Type II importer (24). However, adaptations made for the small size of molybdate allowed previously unseen mechanistic details when MolBC-A was studied in detergent micelles (4). Interestingly, *H. influenzae* is thought to have two molybdate transport systems: the Mol system, which is the focus of this work, and the Mod system (22, 25). Two molybdate transport systems raise interesting questions about the role of a duplicate transport system on substrate uptake and homeostasis in the cell.

To explore the mechanism and function of MolBC-A, we employed techniques that maintained the transporter in a lipid environment. Continuous wave-electron paramagnetic resonance (CW-EPR) spectroscopy was used to probe the mechanism of MolBC in proteoliposomes and determine how the transmembrane helices reposition themselves in response to nucleotide binding and hydrolysis. The function of MolBC-A was studied *in vivo* by monitoring molybdate uptake and transporter expression. Our studies address the impact lipid environment has on the mechanism of MolBC-A as well as the role this transporter plays in molybdate uptake.

## EXPERIMENTAL PROCEDURES

### 

#### 

##### Mutagenesis of H. influenzae

An Rd mutant strain in which HI1469 to HI1473 were deleted was generated using a modification of the recombineering method developed for *Haemophilus* (26). Briefly, HI1469 to HI1473 plus ∼1 kb of genomic DNA was PCR-amplified from strain Rd using Rdmol_cloneF1 and Rdmol_cloneR1 (Table 1), ligated with the Copy Control plasmid, pCC1 (Epicenter, Madison WI), then transformed first into *Escherichia coli* strain EPI300 then into the recombineering strain of *E. coli*, DY380. Bipartite PCR primers (Table 1) were used to amplify a cassette that contained a spectinomycin resistance gene and *sacB* flanked by FLP recognition target (FRT) sites (specR-*sacB*).^3^ The first primer, Rdmol_recombF1, had 20 bp of homology to the P1 binding site adjacent to the FRT site downstream of *sacB* and 50 bp homologous to 47 bp of the intergenic region directly upstream of HI1473 followed by the HI1473 start codon. The second primer, Rdmol_recombR1, had 20 bp of homology to the P2 binding site adjacent to the FRT site 5′ downstream of the spectinomycin resistance gene and 50 bp homologous to the 3′ 36 bp of HI1469 as well as the adjacent 3′ intergenic region downstream of HI1469. The resulting PCR product was transformed into DY380(pCCI-*mol*), and recombination of the specR-*sacB*-containing PCR product into the pCCI-*mol* construct was initiated by incubation at 42 °C for 15 min. The *mol* gene cluster was thus deleted. The Δ*mol*::specR-*sacB* sequence plus the ∼1-kb flanking DNA was then amplified by PCR and transformed into strain Rd using the MIV method (see Refs. 43 and 44). Mutants in which the *mol* gene cluster was deleted via homologous recombination of the Δ*mol*::specR-*sacB* construct were selected on medium containing 50 mg of spectinomycin/ml. One mutant was transformed with a temperature-sensitive derivative of pLS88, which contains both a kanamycin resistance gene and the FLP recombinase gene under control of the *tet* operator/repressor. The cells were grown to mid-log at 32 °C, treated with 200 ng of anhydrotetracycline/ml for 2 h, then plated on non-selective agar and incubated overnight at 37 °C. Induction of FLP recombinase expression allows recombination between the FRT sites, which removed the specR-*sacB* cassette. Incubation at 37 °C facilitated the loss of the temperature-sensitive plasmid. Colonies were screened for the loss of both spectinomycin and kanamycin resistance, which indicated the loss of both the specR-*sacB* cassette and the temperature-sensitive plasmid. A Rd mutant strain was, therefore, isolated that contained a deletion of the *mol* gene cluster and replacement with a FLP scar that contained a small ORF containing the start codon of HI1473, an FRT site, and the last 12 codons of HI1469. Strain RdΔ*mol* mutants were identified by colony PCR using Rdmol_testF1 and Rdmol_testR1, and one was confirmed by sequencing.

**TABLE 1:** **Primer table**

Primer name	Sequence
Rdmol_cloneF1	TTTAAATTCTTAGTTTTTCTGGTTCA
Rdmol_cloneR1	ATTCTTCCGCTTGTTCTATGAG
Rdmol_recombF1	GTAAGCAGATTCAGTTATATCAGGGTCAATTAATTTAAGGAGTTACGATGATTCCGGGGATCCGTCGACC
Rdmol_recombR1	TTGCTGCTTTTTTTTCAGTACTTAGAGACATTTTTTACTCCTAAAAAAGATGTAGGCTGGAGCTGCTTCG
Rdmol_testF2	CGAAAATGCTCAAAACCTACA
Rdmol_testR2	CATAACCGTTGGTCCATTGTA
molB_F	CATTCCTCATCTTAGCCG
molB_R	CATATAAGTCGCCCCAAC
modB_F	AGTGTTGCAGTCAGTTCC
modB_R	CAGGTGGCAACACTAAAG
gyrA_F	ACGGCTTGCTTGTGGAAGTT
gyrA_R	ACGTGGTTGTGACGCTTTCTC

##### Growth of H. influenzae

Rd and RdΔ*mol* strains were grown from backstocks on Chocolate Agar II plates (Fisher: B21169X), then inoculated into 200 ml of Coleman's media (27) and grown at 37 °C for 16 h. When precultured *A*_600_ reached ∼0.6, cells were diluted into 2 liters of Coleman's media to give an *A*_600_ of ∼0.01. The 2-liter cultures were grown in unbaffled 4-liter Erlenmeyer flasks at 37 °C with shaking at 180 rpm. At the time points indicated in Fig. 4, aliquots were removed for real time-quantitative PCR (RT-qPCR) and inductively coupled plasma-mass spectrometry (ICP-MS), and at one point indicated in Fig. 4, sodium molybdate was added to a final concentration of 0.1 mm.

For RT-qPCR, 4 × 10-ml aliquots were removed at the indicated time points and lysed in 1 or 2 ml of TRIzol (Invitrogen; 15596018). Cells were incubated in TRIzol for 5 min then stored at −80 °C. For ICP-MS, 4 × 200-ml aliquots were removed at an *A*_600_ of ∼0.15, whereas 4 × 30 ml aliquots were removed at the other time points. Cells were washed twice in 25 mm Tris, pH 7.5, 100 mm NaCl then stored at −80 °C.

##### RNA Purification and DNase Treatment

RNA was purified with TRIzol according to the manufacturer's protocol. Briefly, RNA was phenol-chloroform-extracted from TRIzol-lysed cell samples, precipitated with isopropyl alcohol, washed with ethanol, air-dried, then resuspended in RNase-free water (Invitrogen; AM9939). To remove contaminating genomic DNA, 10–40 μg of RNA was treated with DNase I (Invitrogen; 18068-015) in the presence of RNase-OUT (Invitrogen; 10777-019) for 1.5 h and terminated with phenol-chloroform (MP Biochemicals; 04802513). RNA was ethanol-precipitated from the upper layer of the phenol-chloroform extraction then washed, air-dried, and resuspended in RNase-free water.

##### cDNA Synthesis and RT-qPCR

cDNA was prepared from 2.2 μg of DNase-treated RNA samples using random hexamer primers and SuperScript III RT (Invitrogen; 18080044) following the manufacturer's instructions. Quantitative-PCR reactions were set up with the above cDNA. For each time point there were four biological replicates (independently purified RNA samples) and two technical replicates. RT-qPCR assays were set up in a 384-well plate (Bio-Rad; HSP-3805) with each 10-μl PCR reaction made up of 10 mm Tris, pH 9.2, 1.5 mm MgCl_2_, 49 mm KCl, 0.2 mm dNTPs, 20 milliunits/μl Platinum-Taq (Invitrogen; 10966–026), 0.1× SYBR Green (Invitrogen; S-7563), 0.125 μm forward primer, 0.125 μm reverse primer, 100× diluted cDNA. Primers molB_F and molB_R were used for the detection of *molB* (HI1471), primers modB_F and modB_R were used for the detection of *modB* (HI1692), and primers gyrA_F and gyrA_B were used for the detection of the gyrase reference gene *gyrA* (Table 1) (28). The PCR reactions were run in a Bio-Rad CFX-384 thermocycler using 40 cycles with an annealing temperature of 52 °C. A melt curve run after the 40th cycle showed that each reaction with a Ct below 25 showed a single peak. A control reaction lacking reverse transcriptase was performed for each RNA sample, and the Ct difference between negative and positive RT reactions was greater than 10 for all analyses. Also the Ct values derived for *molB* in RdΔ*mol* were nearly identical to the Ct values generated with the -RT control samples (Ct > 32). -Fold change was calculated using the ΔΔCt method (29).

##### ICP-MS

Frozen cell pellets were lyophilized for at least 48 h to determine the dry weight. Then cells were resuspended in 25 mm Tris, pH 7.5, 100 mm NaCl, and 0.4–3.2 mg were digested in nitric acid at 65 °C overnight. An internal standard was then added (5 ng/μl ^112^In), and the samples were diluted to 3 ml with metal-free water. The final nitric acid concentration was 3%. Digested samples were analyzed via ICP-MS (Thermo Fisher X Series II) to quantify ^95^Mo concentration by comparison to a molybdenum standard curve.

##### Lipid Preparation

Chloroform-dissolved *E. coli* Polar lipid (Avanti: 100600C) and egg PC (Avanti: 840051C) were mixed in a 3:1 (wt:wt) ratio then dried under a stream of argon. Residual chloroform was removed under vacuum overnight. Lipids were resuspended to 50 mg/ml in 25 mm Tris, pH 7.5, 100 mm NaCl and vortexed to produce a cloudy lipid suspension. Using a micro-tip probe sonicator, lipids were sonicated 10 s on/10 s off for a total on time of 5 min (amplitude = 40). Liposomes were kept covered on ice and used within a few hours of sonication.

##### Protein Reconstitution

The MolBC transporter was purified and spin-labeled as described in Rice *et al.* (4). Liposomes were dissolved with 80 mm sodium cholate. This is the minimum concentration of detergent required to completely dissolve the *E. coli* Polar: egg PC liposomes as verified by an optical density measurement. The dissolved lipid and purified protein were mixed in a 10:1 (wt:wt) ratio and incubated on ice for 5 min. Methanol-activated and buffer-washed biobeads (2/3 the sample volume) were then added to the detergent-lipid-protein samples. Samples were incubated at 4 °C for ∼16 h with rocking, then at room temperature for 2–3 h. Samples were diluted with 25 mm Tris, pH 7.5, 100 mm NaCl to fill a Ti 45 rotor centrifuge tube (Beckman; 355622, ∼73 ml) then spun at 150,000 × *g* for 2 h at 4 °C. The pellet was resuspended in a minimal volume of 25 mm Tris, pH 7.5, 100 mm NaCl.

##### Assay of ATP Hydrolysis

An enzyme-linked inorganic phosphate assay (Cytoskeleton Inc) was used to assay rates of ATP hydrolysis. Assays were set up in a UV transparent 96-well, half-area plate with 0.2 mm 2-amino-6-mercapto-7-methylpurine riboside, 0.1 unit of purine nucleoside phosphorylase, 1 mm MgCl_2_, and MolBC. Each assay was 100 μl, buffered with 25 mm Tris, pH 7.5, 100 mm NaCl. 10–50 μg/ml of MolBC (depending on activity of the mutant) was assayed in triplicate. The assay was started by the addition of 2 mm ATP. The initial rates of *A*_360_ increase for replicate samples were compared to determine standard error of the activity. The length of the initial region was varied to minimize standard error while maintaining a high (>0.98) *r*^2^ factor in the linear regression. A standard phosphate curve was used to quantify activity from the assay results. For specific activity calculations, the protein concentration was determined from the CW-EPR data by comparison of the spin concentration (from integrated absorbance peak compared with a 100 μm MTSL (*S*-(2,2,5,5-tetramethyl-2,5-dihydro-1H-pyrrol-3-yl)methyl methanesulfonothioate) standard) to the spin-labeling efficiency.

##### Continuous Wave-Electron Paramagnetic Resonance

CW-EPR spectra were measured as described previously (4, 9). Briefly, the scan width of all spectra was 300 G, and presented spectra were cropped to values stated in the figure legends. Ligands used for CW-EPR spectroscopy were as follows: MolA at a 2× molar excess over MolBC; 10 mm ATP + 1.5 mm EDTA; 16.5 mm MgCl_2_. Ligands were serially added to recovered protein samples then incubated for at least 10 min (60 min for MolA) before being re-scanned to allow ample time for each sample to hydrolyze the given amount of ATP and reach an equilibrium. CW-EPR spectra were collected at room temperature (23 °C). For spectra showing coupled spins, Gaussian-fitted distance distributions were calculated using the Short Distances program written by C. Altenbach (30).

## RESULTS

### 

#### 

##### Mechanistic Studies of Liposome-reconstituted MolBC Transporter

To understand the transport mechanism, we first had to establish that the effects of substrate binding protein and nucleotide binding on MolBC were conserved in a lipid environment. We used site-directed CW-EPR to study the mechanism of MolBC. With this technique, sites of interest were selectively mutated to cysteine for the covalent attachment of a stable free-radical spin-label, MTSL. Site-directed EPR spectroscopy is particularly sensitive to changes in the motility of the spin-label (and the protein to which it is attached) as well as the distance between spin labels. The sites chosen for this study were on the two gates in MolB that facilitate the passage of molybdate through the transporter: the periplasmic gate and the cytoplasmic gate (4). The translocation pathway of MolBC lies at the interface of the MolB homodimer (helix 5). The periplasmic gate is made up of a small helix (helix 5a) and a loop on the periplasmic side of the transporter. The cytoplasmic gate is made up of a long loop between helices 2 and 3 and is homologous to cytoplasmic gate II identified in BtuCD (31).

##### Periplasmic Gate Movements in a Lipid Environment

**FIGURE 1.**
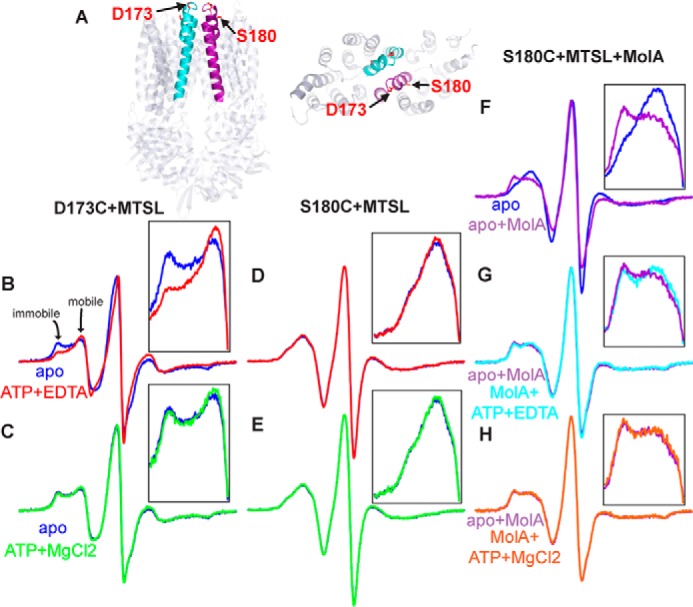
**CW-EPR spectroscopy of TM5a residues showing nucleotide-dependent mobility changes at the periplasmic gate.** *A*, schematic representation of the cytoplasmic gate as viewed from the side and periplasm of MolBC (PDB code 2NQ2) (17). MolB_D173C and S180C (shown as *red sticks*) were spin-labeled with MTSL. TM5 and 5a are colored *cyan* or *purple* according to transmembrane domain monomers. Shown are CW-EPR spectra for D173C+MTSL (*B*) and S180C+MTSL (*D*) (100-G scan width), recorded apo- and ATP-bound (*blue* and *red lines*, respectively). Mobile and immobile components are identified for the S173C spectra. Post-hydrolysis spectra (*green*) of D173C (*C*) and S180C (*E*) overlaid with respective apo spectra are shown. *F*, effect of MolA binding to MolBC_S180C (*purple*) overlaid with an apo spectrum (*blue*). *G*, ATP-bound spectrum of the MolBC-A complex (*cyan*) overlaid with the apo MolBC-A spectrum. *H*, post-hydrolysis spectrum (*orange*) of MolBC-A complex. Overlaid spectra have been normalized by the central peak height. MolBC diagrams were prepared using program PyMOL All protein structure graphics were made with PyMOL (45). Spectra and charts were graphed using Grapher 9 software (Golden Software Inc., Golden, CO).

To provide a lipid environment for CW-EPR spectroscopy analysis, MolBC was reconstituted into proteoliposomes. Fig. 1*A* shows the transporter in the nucleotide-free conformation with the periplasmic gate closed. To observe changes at the periplasmic gate, we probed Helix 5a and the adjacent loop at S180C and D173C, respectively. For D173C, spin-label mobility increases when MolBC binds nucleotide (Fig. 1*B*). The addition of magnesium allows for ATP hydrolysis and the return to a state similar to apo (Fig. 1*C*). The ability of MolBC_D173C to bind and hydrolyze ATP was expected from ATP hydrolysis assay (Table 2). Greater spin-label mobility at D173C in the presence of ATP was also observed in detergent (4), which suggests a similar conformation change at this site in both the lipid and a detergent environment.

**TABLE 2:** **ATP hydrolysis activity for MolBC in liposomes**

MolBC Mutant	Lipid	Specific activity	Standard error of activity
		*nmol P_i_/mg enzyme/min*	*nmol P_i_/mg enzyme/min*
D173C+MTSL	*E. coli* polar:egg PC (3:1)	239	10
S180C+MTSL	*E. coli* polar:egg PC (3:1)	51	3
N93C+MTSL	*E. coli* polar:egg PC (3:1)	415	6

Unlike the results in detergent, the mobility at S180C is not affected by the addition of nucleotide or magnesium (Fig. 1, *D* and *E*, respectively), but conformational changes at S180C may be observed when lipidated MolBC is bound to MolA. When MolBC_S180C forms a complex with substrate-free MolA, a notable decrease in spin-label mobility is observed (Fig. 1*F*). Substrate-free MolA was used for this study because previous studies have shown the presence of molybdate weakened the interaction between MolA and MolBC (32). A slight increase in mobility is observed at S180C when the MolBC-A complex is bound to ATP (Fig. 1*G*) and returns to the apo-like mobility post-hydrolysis (Fig. 1*H*). The diminished effect of ATP at this site (relative to the detergent stabilized MolBC_S180C) may be due to lower affinity of MolBC for ATP. This explanation is supported by the very low ATP hydrolysis rate for this mutant when reconstituted into liposomes.

##### Cytoplasmic Gate Movement in a Lipid Environment

**FIGURE 2.**
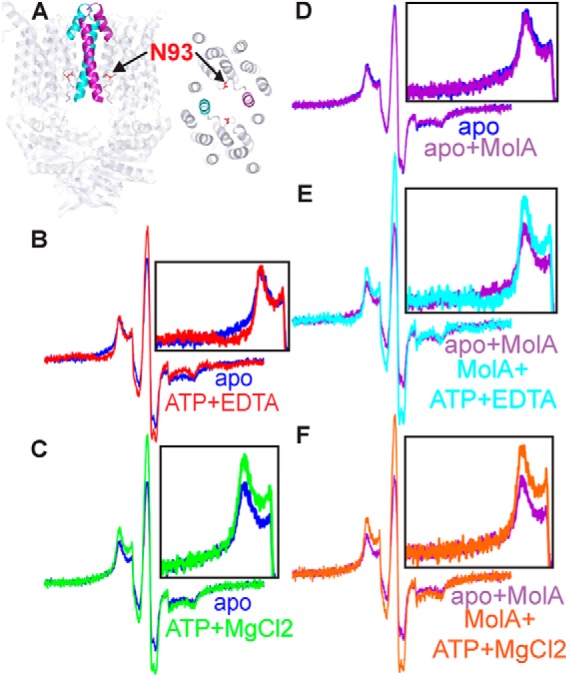
**Nucleotide-dependent movement of the cytoplasmic gate shown by CW-EPR.** *A*, schematic representation of the cytoplasmic gate as viewed from the side and periplasm of MolBC (PDB code 2NQ2) (17). MolB_N93C (shown as *red sticks*) was spin-labeled with MTSL. *B*, CW-EPR spectra for N93C+MTSL (200G). All spectra were normalized for equal spin (normalized double integration values). The apo (*blue*) and ATP-bound (*red*) spectra are overlaid to highlight the broadening of the ATP-bound spectrum, indicative of spin coupling. *C*, the line shape returns to an apo-like state upon the addition of MgCl_2_ (*green*). *D*, effect of MolA binding to MolBC_N93C (*purple*) overlaid with apo spectrum (*blue*). *E*, ATP-bound spectrum of the MolBC-A complex (*cyan*) overlaid with the apoMolBC-A spectrum (*purple*). *F*, post-hydrolysis spectrum (*orange*) of MolBC-A complex overlaid with the apoMolBC-A spectrum (*purple*). All spectra were normalized for equal spin (normalized double integration values). MolBC diagrams were prepared using program PyMOL.

**FIGURE 3.**
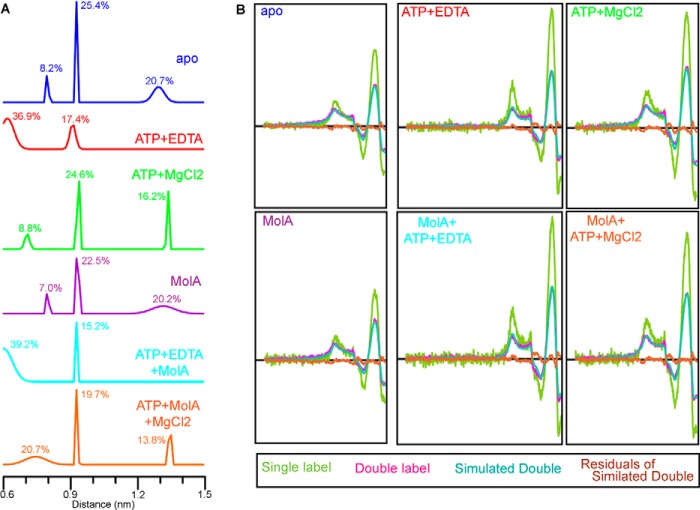
**Analysis of CW-EPR spectroscopy for the cytoplasmic gate.** *A*, distance distributions calculated from CW-EPR spectra for N93C. Data were collected at room temp (296K) with shown ligands. *B*, single-labeled samples of MolBC_N93C (*lime-green spectra*) were analyzed to facilitate quantitative distance measurement of double-labeled samples (*magenta*). Base-line-corrected double-labeled spectra were iteratively fit with spectra simulated from Gaussian distance distributions (*aqua green*) using the short distances program (30). The residuals of the simulated spectra (*brown*) can be compared with the zero axis (*black*) to estimate goodness of fit. Non-interacting spin for apo, ATP-bound, ATP+Mg^2+^, MolA-bound, ATP+MolA-bound, ATP+Mg^2+^+MolA-bound samples was calculated at 57.8, 53.6, 57.2, 57.3, 56.0, and 53.2% respectively. Respective χ^2^ values for the fitted spectra are 1.43 × 10^−9^, 1.95 × 10^−9^, 2.33 × 10^−9^, 2.02 × 10^−9^, 2.83 × 10^−9^, and 2.11 × 10^−9^.

The cytoplasmic gate was probed at N93C (Fig. 2*A*). With the addition of ATP, the appearance of low-field absorbance in the EPR spectrum indicates that the cytoplasmic gate closes in the ATP-bound state (Fig. 2*B*). As in detergent, this broadened feature of the spectrum relates to a very close distance between spin labels (Fig. 3). ATP hydrolysis rates in MolBC_N93C are relatively high (Table 2), and the addition of Mg^2+^ enables the shift to an apo-like post-hydrolysis state (Fig. 2*C*). When the experiments are repeated in the presence of MolA-binding protein for a MolBC-A complex, the cytoplasmic gate assumes the same slightly open conformation observed with the MolBC transporter (Fig. 2*D*). Similar to in-detergent experiments, MolA does not appear to affect the cytoplasmic gate conformation in the ATP-bound state (Fig. 2*E*) or the post-hydrolysis state (Fig. 2*F*).

##### Functional Studies of in Vivo Molybdate Transport

**FIGURE 4.**
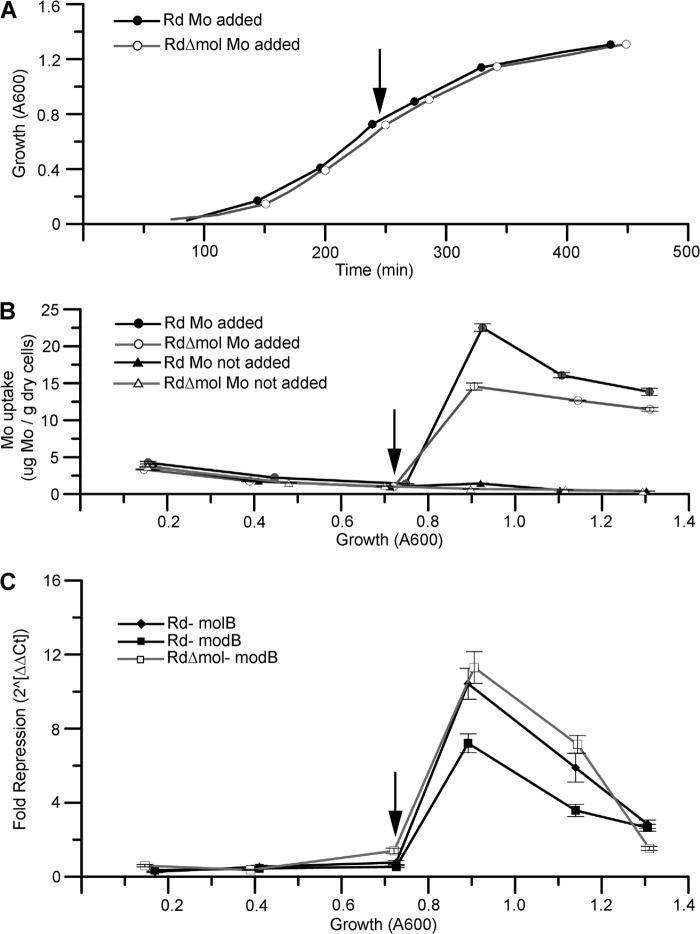
**Growth and analysis of Rd and RdΔ*mol*.** *A*, sigmoidal growth curves of Rd (●) and RdΔ*mol* (○) grown in Coleman's medium. The point of molybdate addition (final concentration of 0.1 mm Na_2_MoO_4_) is marked with a *black arrow. B*, molybdate uptake of Rd and RdΔ*mol* with molybdate added (● and ○) and without added molybdate (▴ and ▵) as measured by ICP-MS. *Error bars* represent the standard error of four independently processed samples from a given growth. *C*, -fold change of *molB* and *modB* repression for Rd parental strain (♦ and ■, respectively) and *modB* for RdΔ*mol* (□). ΔΔCt -fold repression represents 2̂(−ΔCt_Mo_not_added_)/2̂(−ΔCt_Mo_added_), and each ΔCt value represents the difference in transcript levels of target to gyrase reference (Ct_target_ − Ct_reference_). *Error bars* represent the standard error propagated from eight independently processed samples, each assayed in duplicate from growth with and without molybdate addition.

*H. influenzae* strains were grown in Coleman's defined medium supplemented with 17.5 μg/ml hemin and 8.7 μg/ml β-NAD (27). Growth of the *mol* deletion strain RdΔ*mol* and the parental strain Rd followed expected sigmoidal growth curves (Fig. 4*A*). Given the cell mass required for ICP-MS analysis, the earliest time point taken was at an *A*_600_ of ∼0.15. Prolonged incubation of cells in stationary phase (an *A*_600_ of ∼1.3) resulted in cell lysis as observed by a drop in optical density (data not shown). For this reason, precultures were harvested at mid-log (an *A*_600_ of ∼0.6), and all growth time courses were terminated when the culture reached an *A*_600_ of ∼1.3. Aliquots for each time point were collected in quadruplicate from a given growth for standard error calculation.

When grown in Coleman's medium without additional molybdate added, both the parental strain and the RdΔ*mol* deletion strain show equivalent levels of molybdate uptake (Fig. 4*B*). During growth, the amount of molybdenum per cell drops exponentially as the medium is depleted of accessible molybdate and the amount taken up early in the growth is shared among subsequent generations. Analysis of the growth medium shows that the concentration of extracellular molybdate drops from 17.34 ± 0.06 to 11.5 ± 0.1 nm, indicating the minimum level of the molybdate scavenging capacity in *H. influenzae* strain Rd. These analyses make it clear that the *mol* deletion did not affect molybdate uptake at low molybdate concentrations.

Previous studies on the affinity of MolA for molybdate indicated that MolBC-A was a low affinity transporter (22). We were thus interested in observing the effects of a mol deletion at higher molybdate concentrations. To test this, we grew cultures to mid-log where molybdate would be largely scavenged from the medium (Fig. 4*B*). We then dosed the culture with 100 μm sodium molybdate and continued to monitor molybdenum uptake and transporter transcription. As anticipated, the parental strain was able to bring in more molybdate at a faster rate than the *mol* deletion strain (Fig. 4*B*). These results indicated that the presence of both molybdate transport systems allowed up to 60% more molybdate to be taken up after the medium was supplemented with molybdate. After peak influx, molybdate levels for the parental strain and RdΔ*mol* settle toward a common value.

Expression levels of the two molybdate transporters were followed via RT-qPCR (targeting *molB* and *modB*, the genes that encode the transporter transmembrane domains). In the parental strain, the addition of molybdate repressed the transcription of *modB* 7-fold and *molB* 10-fold when comparing samples taken at an *A*_600_ of 0.9 in either the presence or absence of molybdate (Fig. 4*C*), indicating that the increase in cytoplasmic molybdate leads to a repression of molybdate transporter transcription. The addition of molybdate likewise repressed the transcription of *modB* 10-fold in the RdΔ*mol* deletion strain (Fig. 4*C*). After an *A*_600_ of 0.9 and the observed peak of repression, the molybdate transporter genes undergo slow de-repression, but they do not reach the level of expression observed during non-supplemented growth.

**FIGURE 5.**
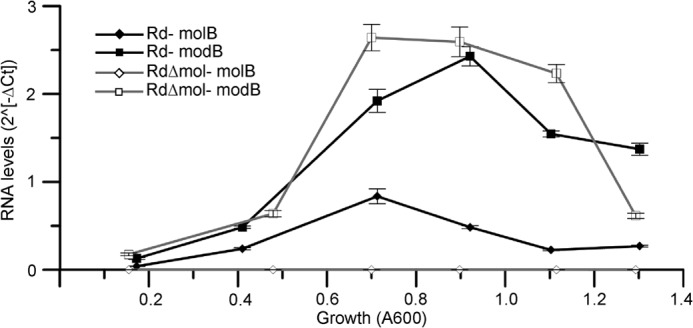
**RT-qPCR analysis of Rd and RdΔ*mol* without added molybdate.** Transcript levels of *molB* and *modB* in Rd (■ and ♦, respectively) and in RdΔ*mol* (□ and ♢, respectively). RT-qPCR data displayed as -fold difference of target gene to gyrase reference gene (2̂(−ΔCt) = 2̂(−(CT_target_ −CT_reference_))). *Error bars* represent the standard error of four independently processed samples from a given growth each assayed in duplicate.

Analysis of the transcript levels during non-supplemented growth shows that the transporters follow a trend of low transcription at low ODs and peak transcription at late log phase, with transcript levels of *modB* consistently greater than *molB* (Fig. 5). These data indicate that transcription of both transporters is minimal when cytoplasmic molybdate concentration is higher, whereas transcription is significantly greater when molybdate concentration is low. This may indicate a repression mechanism at high concentrations of cytoplasmic molybdate. As expected, the transcription of *molB* is eliminated in RdΔ*mol*. Interestingly, transcript levels of *modB* were similar in the parental strain and RdΔ*mol* (Fig. 5), suggesting that the *mol* deletion had little or no direct effect on *modB* expression. In late log phase there appears to be a drop in transporter expression that is not correlated to molybdate concentration. This drop in expression may be due to the culture reaching stationary phase and may contribute to the apparent de-repression observed in Fig. 4*C*.

## DISCUSSION

To decipher the effects of a lipid environment on the mechanism of MolBC-A, the transporter was reconstituted in proteoliposomes and analyzed by site-directed CW-EPR. A lipid bilayer appears to have interesting effects on conformational changes at different sites of MolBC-A, but the mechanism of MolBC is generally conserved.

In lipids, the periplasmic gate appears to open when MolBC binds nucleotide, which would allow substrate to pass from the periplasmic-binding protein MolA to the translocation pathway. An increase in mobility, representing the putative substrate-permeable state, was observed at D173C in the loop adjacent to helix 5a. This movement at the periplasmic gate is akin to the unlocking gate mechanism observed in detergent (4). Likewise, a nucleotide-dependent increase in mobility was observed at S180C in the presence of MolA, but this conformation shift at helix 5a represented a small fraction of the transporters. Overall, the conformation of the periplasmic gate appears to be more restricted in a lipid environment than in detergent. At the cytoplasmic gate, nucleotide closes the distance between spin-labels independent of MolA. The closed cytoplasmic gate reopens after ATP hydrolysis, which would allow substrate to pass from the translocation pathway into the cytoplasm. At this site the most notable effect of lipid appears to be an increase in immobile spin label, which is most likely due to a more restricted conformation at the cytoplasmic side of the translocation pathway.

The presence of lipid also has a notable effect on activity; with one exception, ATP hydrolysis activities are reduced 4–50-fold depending on the mutant assayed (Table 2). This impact of the lipid bilayer on transporter activity was anticipated as basal activity of MalFGK decreased in a more native, lipid environment (8). The one exceptional case was MolBC_D173C, which had abnormally low activity in detergent (4) and when reconstituted showed a slight increase in activity. Reconstituted MolBC_D173C shows similar levels of activity to other reconstituted mutants, which is lower than the typical MolBC activity in detergent. Vigonsky *et al.* (32) have suggested that low levels of basal ATP hydrolysis in the presence of molybdate may serve to disassociate MolA from the stable MolBC-A complex and so restart the transport cycle.

Our *in vivo* studies allow MolBC-A to function in a nearly native environment with controlled growth conditions. In this system MolBC-A is embedded in native lipids, regulated by native systems, and imports substrate in the presence of the *H. influenzae* molybdate transporter ModBC-A. By comparing the *mol* deletion to its parental strain, we see that MolBC-A allows the bacteria to take up more molybdate at a quicker rate. Because a disadvantage in the deletion strain is not observed at low molybdate concentrations, we may conclude that ModBC functions primarily during periods of low external molybdate, whereas MolBC functions at high molybdate concentrations. This role of MolBC-A was predicted by studies of MolA, which showed it to have lower affinity for substrate than a typical ModA-binding protein (22). The expression of MolBC-A at low molybdate concentrations may allow for limited molybdate transport. However, it seems more likely that MolBC-A expression at low molybdate levels would prepare the cell for a brief increase of molybdate in the growth environment.

**FIGURE 6.**
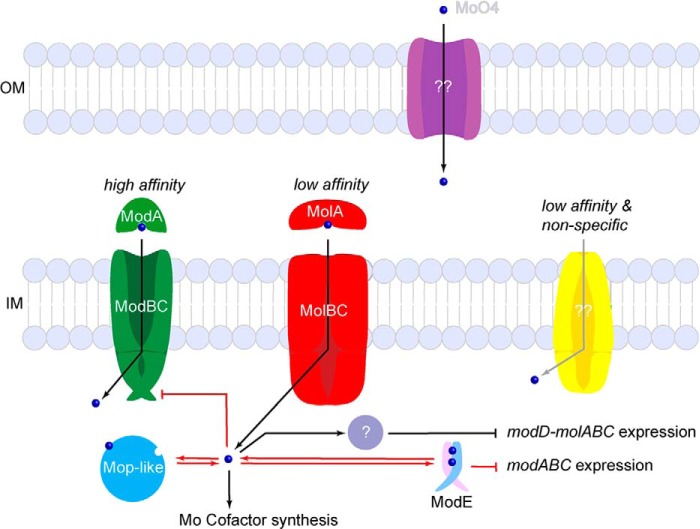
**Putative overview of molybdate uptake in *H. influenzae*.** Once molybdate passes into the periplasm, it can be taken into the cytoplasm by three transport systems: the high affinity system (ModBC-A), the low affinity system (MolBC-A), or the nonspecific low affinity system (observed in *E. coli*). Cytoplasmic molybdate can be used for molybdenum cofactor synthesis, sequestered by Mop-like proteins, or used to down-regulate additional molybdate uptake. Pathways known to exist in *H. influenzae* are marked with *arrows*; components marked in *red* were identified in alternate bacteria with homologous systems. (*OM*, outer membrane; *IM*, inner membrane.)

The *H. influenzae* system of high and low affinity molybdate transporters has precedent in other bacteria. Fig. 6 depicts our current understanding of molybdate uptake with contributions from the present studies on *H. influenzae* (*black text* and *arrows*) and studies with other bacteria (*red*) (33–37). Work with *E. coli* has revealed three transporters capable of molybdate uptake: 1) a high affinity ModBC-A homolog of the same name; 2) a low affinity sulfate/molybdate transporter CysUWA-substrate-binding protein; 3) an unidentified, low affinity, nonspecific importer (34, 35). Although *H. influenzae* MolA specifically binds molybdate (and tungstate) (22), the two low affinity *E. coli* transporters may play a homologous role to MolBC-A. Once in the periplasm of *H. influenzae*, active transport of molybdate to the cytoplasm may occur through ModBC (by a mechanism well described in MalFGK (38, 39)), MolBC, or a putative nonspecific transporter (Fig. 6).

In *E. coli*, cytoplasmic molybdate is sequestered by Mop-like proteins (33) used for molybdenum cofactor synthesis or down-regulates the uptake of additional molybdate. Two mechanisms of ModBC regulation are known: direct inhibition by molybdate binding to the ModC regulatory domain as in *Methanosarcina acetivorans* (36) and repression of *modABC* expression by molybdate-binding ModE (37). The *H. influenzae* ModC is predicted to have a C-terminal TOBE or regulatory domain (exon.niaid.nih.gov), and a ModE homolog has been identified in this organism (33). Regulation of MolBC-A transport is poorly understood, but our present work shows that despite an extracellular molybdate concentration of 100 μm, intracellular levels accumulate no higher than 22 μm, suggesting that a point is reached where even the low affinity transporters are regulated (intracellular molybdate concentration calculated assuming that *H. influenzae* had a similar volume and dry weight as *E. coli* (40–42)). The molybdate-dependent repression of *molB* suggests the presence of an unknown factor to couple cytoplasmic molybdate concentration to the transcription of the *molABC* operon. The period of growth when ModBC is inhibited but MolBC is still active may explain why the parental strain was able to take up more molybdate than the *mol* deletion strain.

Combined, these results reveal new details about bacterial transport and the role of MolBC-A in molybdate uptake. When studied in lipid, the nucleotide-dependent conformation shifts of MolBC confirm our previously described mechanism (4). A lipid environment decreases the measured activity of MolBC as well as the conformational flexibility of the regions studied. However, the gate movements that facilitate transport are still allowed. When molybdate transport was studied *in vivo*, we were able to add context to the transport model. In *H. influenzae*, MolBC-A is the low affinity molybdate transporter. It allows the pathogen to quickly take advantage of high concentrations of extracellular molybdate. However, because of the abundance and diversity of transporters per organism, understanding the coordination of low and high affinity transport activities is in its infancy. Additional investigation focusing on transport regulation will be needed to understand how multiple transport systems function concertedly in bacterial transport.

## Footnotes

3 Holland, I. B. (2003) *ABC Proteins: From Bacteria to Man*, pp. 187–207, Academic Press, San Diego, CA.

## Footnotes

2 The abbreviations used are:

ABC: ATP binding cassette
CW-EPR: continuous wave-electron paramagnetic resonance
ICP-MS: inductively coupled plasma-mass spectrometry
MTSL: *S*-(2,2,5,5-tetramethyl-2,5-dihydro-1H-pyrrol-3-yl) methylmethanesulfonothioate
qPCR: quantitative PCR
FRT: FLP recognition target
PC: phosphatidylcholine.
